# A mini review on aflatoxin exposure in Malaysia: past, present and future

**DOI:** 10.3389/fmicb.2013.00334

**Published:** 2013-11-13

**Authors:** Sabran Mohd-Redzwan, Rosita Jamaluddin, Mohd Sokhini Abd.-Mutalib, Zuraini Ahmad

**Affiliations:** ^1^Department of Nutrition and Dietetics, Faculty of Medicine and Health Sciences, Universiti Putra MalaysiaSerdang, Malaysia; ^2^Department of Biomedical Sciences, Faculty of Medicine and Health Sciences, Universiti Putra MalaysiaSerdang, Malaysia

**Keywords:** aflatoxin, aflatoxin B_1_, aflatoxin exposure, Malaysia, Southeast Asia

## Abstract

This mini review article described the exposure of aflatoxin in Malaysia, including its presence in the foodstuffs and the detection of aflatoxin biomarkers in human biological samples. Historically, the exposure of aflatoxin in Malaysia can be dated in 1960s where an outbreak of disease in pig farms caused severe liver damage to the animals. Later, an aflatoxicosis case in Perak in 1988 was reported and caused death to 13 children, as up to 3 mg of aflatoxin was present in a single serving of contaminated noodles. Since then, extensive research on aflatoxin has been conducted in Malaysia. The food commodities such as peanuts, cereals, spices, and their products are the main commodities commonly found to be contaminated with aflatoxin. Surprisingly, some of the contaminated foods had levels greater than the permissible limit adopted by the Malaysian Food Regulation 1985. Besides, exposure assessment through the measurement of aflatoxin biomarkers in human biological samples is still in its infancy stage. Nevertheless, some studies had reported the presence of these biomarkers. In fact, it is postulated that Malaysians are moderately exposed to aflatoxin compared to those high risk populations, where aflatoxin contamination in the diets is prevalent. Since the ingestion of aflatoxin could be the integral to the development of liver cancer, the incidence of cancer attributable by dietary aflatoxin exposure in Malaysia has also been reported and published in the literatures. Regardless of these findings, the more important task is to monitor and control humans from being exposed to aflatoxin. The enforcement of law is insufficient to minimize human exposure to aflatoxin. Preventive strategies include agricultural, dietary, and clinical measures should be implemented. With the current research on aflatoxin in Malaysia, a global networking for research collaboration is needed to expand the knowledge and disseminate the information to the global scientific community.

## INTRODUCTION

There are many toxic compounds produced by fungi and one of them is mycotoxin. The occurrence of mycotoxin in the food commodities can be dated as early as in the tenth century. The infection of fungi in the diets caused the outbreak of disease known as St. Anthony’s or Holy Fire in many European countries (Paterson and Lima, 2010). Paterson and Lima (2010) reported the outbreak was intensified with the contamination of rye by ergot alkaloid, produced by *Claviceps purpurea*. Since then, many cases have been reported and the discovery of “Turkey X” disease caused by aflatoxin, in 1950s and early 1960s had opened new prospectus on the scientific research on the etiology of mycotoxicosis and preventive strategies in foods, animals, and humans (Kensler et al., 2011). Kensler et al. (2011) described the epidemic disease as the major cause of death of numerous poultry animals including ducklings and chicks due to the consumption of diet containing contaminated peanuts. Further investigations at that time revealed the toxicity was associated with *Aspergillus flavus*, a pathogenic fungus and extracts from the culture of the fungus isolated from the meal were found to have the capability to induce the “Turkey X” syndromes (Kensler et al., 2011). Due to this, most of the reported cases on mycotoxicosis focused on specific species of fungi that are found in many contaminated foods and feeds. Indeed, four major species of fungi have been discovered belonging to the species of *Aspergillus*, *Fusarium*, *Penicillium*, and *Claviceps* that produced some major mycotoxins such as aflatoxin, ochratoxin A, fumonisim, and zearalenone (Paterson and Lima, 2010). Of these four mycotoxins, research on aflatoxin has been extensively conducted as aflatoxin represents the global main threat due to its toxicity (Paterson and Lima, 2010; Kensler et al., 2011)

Aflatoxin is produced by *A. flavus*, *A. paraciticus*, and *A. nomius* and ubiquitously found in foodstuffs (Reddy et al., 2011). Since aflatoxins are visible under ultraviolet (UV) light, the first attempt to detect aflatoxin is mainly based on this criteria (Groopman et al., 2005). Later the isolation of purified aflatoxin metabolites with identical physical and chemical properties formed the core to the scientific research on aflatoxin (Kensler et al., 2011). These findings have stimulated numerous research efforts, which transcend to the present, to assess possible hazards of aflatoxin contamination in the human food sources and finally to reduce the exposure through various preventive strategies. In one of many instances, the development of analytical methods to detect and quantify aflatoxin in foods and feeds is significance (Kensler et al., 2011) as more studies are able to be conducted to determine the association of aflatoxin ingestion with diseases in human population, especially with the incidence of hepatocellular carcinoma (HCC). Moreover, as the science is moving forward in parallel to the advancement of technology, the development of method for structural characterization, and synthesis of the major aflatoxins has led to better understanding of the mechanistic studies of their toxicology and metabolism (Groopman et al., 2005; Paterson and Lima, 2010; Kensler et al., 2011). For instance, the isolation of aflatoxin biomarkers in human biological samples such as serum AFB_1_-DNA adduct, AFB_1_-lysine adduct, and other metabolites of AFB_1_ in urine and feces (Wang et al., 1999; Mykkänen et al., 2005; Polychronaki et al., 2008) can give a better indication on the extent of human exposure to aflatoxin. These data are essential for the risk assessment as more concrete evidence can be used to evaluate human exposure to aflatoxin in a population.

## PAST-HISTORY OF AFLATOXIN IN MALAYSIA

Malaysia is one of the Southeast Asia countries and geographically located in the equatorial area. Due to that, Malaysia experiences tropical climate with high temperature around 28–31°C (Abdullah et al., 1998). On average, the relative humidity ranged from 70 to 80% during wet season and 50–60% during the dry season (Sulaiman et al., 2007). Furthermore, commodities stored under these conditions are easily deteriorated and susceptible to fungal infections (Abdullah et al., 1998; Sulaiman et al., 2007). With these conditions, the most prominent contamination is the infection of foods by mycotoxin-producing fungi. Aflatoxin, one of the mycotoxin is produced by *Aspergillus* species of fungi and this toxin has been classified by the International Agency for Research on Cancer (IARC) as Group 1 carcinogen and is linked to the development of liver cancer (International Agency for Research on Cancer [IARC], 1993).

In Malaysia, the first indicator of problem associated with aflatoxin occurred around 1960s due to the outbreak of disease in two pig farms in Malacca (Hamid, 1997). Lim and Yeap (1966) reported the presence of aflatoxin in the feed ingredients, supported the evidences of severe liver damage to the animals. However, an episode in Perak, Malaysia around 1988 reminded the hazard of aflatoxin to human health, as the public was shocked by the death of 13 children as a result of consuming contaminated noodles called *loh shi fun*. The post-mortem was performed and the cause of death was associated with acute hepatic encephalopathy (Lye et al., 1995). The culprit was aflatoxin as Lye et al. (1995) reported up to 3mg of aflatoxin was detected in a single serving of *loh shi fun*. Furthermore, the wheat flour used to make the noodles was reported to contain aflatoxin due to the poor storage and processing, which in turn promoted the growth of pathogenic *Aspergillus* fungi and subsequently the production of aflatoxin. Since then, extensive research on aflatoxin has been carried out and the first monitoring was performed by the Institute of Medical Research (IMR), followed by other agencies such as The Food Technology Center (FTC), MARDI, and local universities (Hamid, 1997). Up till know, various studies in Malaysia have reported the presence of aflatoxin in the foodstuffs. Moreover, the biomarkers of aflatoxin have also been reported recently in serum and urine samples, which can provide additional information for assessing aflatoxin exposure in Malaysia.

## PRESENT – OCCURRENCE OF AFLATOXIN IN THE FOOD COMMODITIES, HUMAN EXPOSURE AND RISK ASSESSMENT

The contamination of foodstuffs by aflatoxin has been documented in the literatures. Peanuts, cereals, spices, and their products are the commodities most susceptible to aflatoxin contamination. These food commodities are generally used in many Malaysian delicacies either as a main ingredient or as a base material (Reddy et al., 2011). In fact, they are available almost in all retail shops in Malaysia and are inexpensive. For example, various studies have detected aflatoxin in the peanuts and peanut-based products as shown **Table 1**. Sulaiman et al. (2007) indicated that raw shelled peanut and its products are the good substrate for the growth of aflatoxin by *A. flavus* and *A. paraciticus.* Even though peanuts are not extensively produced in Malaysia, the occurrence of aflatoxin in this food commodity is frequently reported. Moreover, Leong et al. (2011) postulated that high level of AFB_1_ in nuts can result in the high potential risk of AFB_1_ exposure in Malaysia. In fact, Malaysians probably consume higher amount of AFB_1_ than Europeans and Americans but lower than those in Africa and China (Leong et al., 2011).

**Table 1:** Occurrence of aflatoxin in the foodstuffs in Malaysia.

Food commodity	Samples	Positive samples, *n* (%)	Aflatoxin levels	Important findings	Reference
Spices and herbs	Whole and ground, black and white peppercorn	70/126 (55.5)	0.1–4.9 ng/g^a^	None of the positive had levels exceeded the regulatory limit set by the Malaysian Food Regulation 1985. Black peppers were more contaminated than white pepper	Jalili et al. (2009)
	Commercial dried chili	52/80 (65.0)	0.2–56.61 ng/g^b^	6 (7.5%) and 9 (11.3%) of positive samples had AFB_1_ and total AFs levels greater than 5 ng/g for AFB_1 _and 10 ng/g for total AFs	Jalili and Jinap (2012)
	Spices from Penang markets	14/15 (93.3%)	0.58–4.64 μg/kg^b^	Two cumin powders contained highest levels of AFB_1_ ranging from 1.89 to 4.64 μg/kg. Besides, four pepper samples were contaminated with AFB_1_ ranging from 0.65 to 2.1 μg/kg	Reddy et al. (2011)
	Chili	9/10 (90.0%)	5.85–44.2 ng/g^a^	Samples were contaminated mainly by AFB_1._ Samples that contained AFB_1_ had levels exceeded the legal limits established by European. In fact, one of the positive samples had AFB_1_ level of 33.2 ng/g, about seven times higher than the regulatory limit of 5 ng/g by Malaysia Food Regulation, 1985	Khayoon et al. (2012)
Cereals	Starch-based foods (wheat flour)	18/83 (21.7)	11.25–436.25 μg/kg^a^	Of 18 positive samples, 11 had total AFs concentration higher than permitted level adopted by the Malaysian Food Regulation 1985 (i.e., 35 μg/kg), ranging from 41.88 to 436.25 μg/kg	Abdullah et al. (1998)
	Rice, wheat, barley, oat, and maize-meal	40/80 (50.0)	0.12–4.42 ng/g^a^	Although the levels detected were not high, the highest level was found in rice samples. Moreover, 60 out of 80 samples analyzed had at least one mycotoxin	Soleimany et al. (2012b)
	Rice, wheat, oat, barley, and maize-meal	60/100 (60.0)	0.12–4.54 ng/g^a^	More than 50% of sample analyzed contained aflatoxin. Two rice samples were contaminated with aflatoxin at levels of 4.32 and 4.54 ng/g. In fact, 77 of 100 cereal samples were detected to have at least one myctoxin	Soleimany et al. (2012a)
	Cereal-based foods	29/45 (64.4)	0.65–8.95 μg/kg^b^	High incidence of AFB_1_ was found in corn-based product (75%), followed by rice-(69.2%), wheat-(64.2%) and oats-(50%) based foods	Reddy et al. (2011)
	Corn-based products	5/11 (45.5%)	2.5–12.2 ppb (AFB_1_), 5.8–101.8 ppb (AFB_2_)	Two popcorn products exceeded the Malaysia permissible level of 5 ppb	Hong et al. (2010)
	Red rice	46/50 (92%)	0.61–77.3 μg/kg^a^	*Aspergillus flavus* was isolated in 44% samples. In addition, 35 red rice samples had AFs level higher than the Malaysian standard of 5 μg/kg and European standard of 4 μg/kg	Samsudin and Abdullah (2013)
	Black and white rice	4/5 (80.0%)	1.10–5.28 ng/g^a^	One black and white rice respectively had total AFs greater than 5 ng/g set by the Malaysia Food Regulation, 1985	Khayoon et al. (2012)
Peanuts and nuts	Peanut and nuts	13/20 (65.0%)	0.66–15.33 μg/kg	Of 13 peanuts samples, 11 were positive with AFB_1_ with the mean contamination of 4.25 μg/kg	Reddy et al. (2011)
	Nuts and commercial nutty products	32/196 (16.3)	16.6–711 μg/kg^a^	The raw groundnut (shell) and coated nut product had total concentration of aflatoxin of 711 and 514 μg/kg, respectively	Leong et al. (2010)
	Raw shelled peanuts	73/145 (50.0)	0.8–762.1 μg/kg^a^	45% of positive samples exceeded the maximum permitted levels of 35 μg/kg adopted by the Malaysian Food Regulation, 1985	Sulaiman et al. (2007)
	Nuts and nut products	73/128 (57.0)	0.4–222 μg/kg^b^	17 of the 128 samples (13.3%) analyzed had AFB_1_ greater than permissible set by the European Commission. The highest mean value of AFB_1_ was detected in fried peanuts (58.9 μg/kg), followed by bakery products (12 μg/kg)	Leong et al. (2011)
	Raw peanut kernels	66/84 (78.6)	2.76–97.28 ng/g^a^	Of these positive samples, 10.7% had levels exceeded the maximum tolerable of 15 ng/g for total aflatoxin set by Codex. AFB_1_ was the most prevalent aflatoxin detected, followed by AFB_2_ and AFG_1_	Arzandeh et al. (2010)
	Peanut	4/9 (44.4%)	2.15–6.36 ng/g^a^	At least one metabolite of aflatoxin was detected in the positive samples, except for AFB_2._ The level of AFB_1_ (6.00 ng/g) was the highest among the positive samples	Khayoon et al. (2012)
	Peanuts-based product	5/9 (55.6%)	19.5–33.4 ppb (AFB_1_), 0.2 ppb (AFB_2_)	AFB_1_ was found predominantly in the positive samples	Hong et al. (2010)

*AFs, Aflatoxins;*

a Total aflatoxin;

b AFB_1_.

Cereals such as rice are one of the main agricultural products in Malaysia. The presence of aflatoxin in the rice could pose serious health issues. For example, in a study by Soleimany et al. (2012a), 33.3% of analyzed rice from the retail market had detectable level of aflatoxin ranging from 0.19 to 3.96ng/g. Even though the levels are not considered dangerous and harmful to humans, it was an unexpected finding as rice is the main staple food consumed by majority of Malaysian. Statistically, Malaysians consume rice about 289.68g/day (Ministry of Health [MOH], 2006). Thus it is assumed that dietary aflatoxin exposure from rice is somewhere between 55.04ng/day and 1.15μg/day [288.68g/day×0.19 or 3.96ng/g] for an adult of 60kg. Given that the aflatoxin-contaminated rice is consumed on a regular basis, this small amount of aflatoxin at the end will be accumulated in the body and can be detrimental to the health as previously reported.

As highlighted in **Table 1**, varieties of food samples were found to be contaminated with aflatoxin. The detection rate ranged from 16% up to 92% showing the ubiquitous presence of aflatoxin in the human food resources. Although the findings do not represent the whole scenario of aflatoxin contamination in Malaysia, such information shows the pervasiveness of human exposure to this food contaminant. In an instance, Reddy et al. (2011) detected aflatoxin in the foods normally consumed by Malaysian, where they are used and consumed on a daily basis. The presence of aflatoxin in the food chain is a serious matter but not knowing its impact to the health is a big problem and should be a public concern. Indeed in Malaysia, a recent survey reported low awareness and knowledge among the public on the problems associated with fungal and aflatoxin contamination in the diets (Mohd Redzwan et al., 2012a). Furthermore, the similar observations were also reported by several studies in African countries (Jolly et al., 2006, 2009; Ilesanmi and Ilesanmi, 2011).

Exposure assessment by measuring aflatoxin levels in the food samples and extrapolating to estimate average intake at the population level is of low reliability (Kensler et al., 2011). The reason is such data cannot provide information on the individual’s exposure as different individuals may have different exposure to aflatoxin. Nevertheless, the expansion of metabolomic study such as the use of aflatoxin biomarkers could provide the tools for molecular epidemiology to evaluate aflatoxin exposure among individuals within human population. Besides, measuring aflatoxin biomarkers could improve estimation of aflatoxin exposure (Liu and Wu, 2010). The aflatoxin biomarkes such as AFB_1_-DNA adduct, AFB_1_-lysine adduct, and urinary AFM_1_ have been documented in the literatures especially in African countries (Gong et al., 2002; Jolly et al., 2006; Shuaib et al., 2010) and China (Mykkänen et al., 2005; Xu et al., 2010), where aflatoxin contaminations are ubiquitous. These biomarkers are useful for epidemiologist and public health workers to make a better postulation on the extent and severity of aflatoxin exposure in a population. For example, the detection of AFB_1_-DNA adduct such as AFB_1_-N^7^-guanine in the urine samples could be an indicator of mutagenic effect of aflatoxin.

In Malaysia, exposure assessment by measuring aflatoxin biomarkers in human biological samples is still in its infancy. Nevertheless, several studies have reported the presence of these biomarkers among the Malaysia population. Indeed, it is believed that study by Zulhabri et al. (2009) is the first one in Malaysia that reported AFB_1_-albumin adduct in the HCC patients. Zulhabri et al. (2009) indicated that the HCC patients had significantly higher level of AFB_1_-albumin adduct compared to the healthy subjects. Aflatoxin can be an integral to the development of liver cancer (International Agency for Research on Cancer [IARC], 1993), thus the finding could provide an interesting insight on the synergistic effect of aflatoxin exposure. On the other hand, a recent study by Leong et al. (2012) found AFB_1_-lysine adduct in 97% of 170 subjects in Penang, with the levels ranging from 0.2 to 23.16pg/mg albumin. The study also reported that Chinese and Indian respectively were 3.05 and 2.35 times more likely to have high AFB_1_-lysine adduct compared to the Malay (Leong et al., 2012). As a matter of fact, it was corroborated with a report from the Ministry of Health, Malaysia that showed high incidence of liver cancer among Chinese compared to other main ethnicities (Zainal Ariffin and Nor Saleha, 2011). Statistically in 2007, the report listed liver cancer as number 5 out of 10 most frequent cancers among male Chinese with the incidence rate of 5.9% (Zainal Ariffin and Nor Saleha, 2011). Besides, the other aflatoxin biomarkers have also been investigated. A preliminary study involving 22 adult subjects found the presence urinary AFM_1_ ranging from 0.029 to 0.15ng/ml (Sabran et al., 2012). Moreover, a significant and positive association was detected between urinary AFM_1_ with the consumption of milk and dairy products among 98 subjects with detectable level of AFM_1_ in Malaysia (Mohd Redzwan et al., 2012b). Since AFM_1_ is a metabolite of AFB_1_, such findings indicated by Mohd Redzwan et al. (2012b) and Sabran et al. (2012) are useful to estimate human exposure to aflatoxin as a study has found good correlation between dietary intake of AFB_1_ and urinary excretion of metabolite AFM_1_ (Zhu et al., 1987).

Given that ingestion of aflatoxin can be linked to the development of liver cancer, a risk assessment can provide additional information on the extent of human exposure to this food contaminant. Risk assessment is the process of estimating the magnitude and the probability of a harmful effect to individuals or populations for certain agents or activities (Liu and Wu, 2010). Several studies have reported Malaysian population could be exposed as high as 140ng/kg body weight/day (**Table 2**). In one of the examples given in **Table 2**, Chin et al. (2012) indicated that Malaysian exposed to AFB_1_ ranging from 24.3 to 34ng/kg body weight/day and such exposure could contribute to 12.4–17.3% of liver cancer in Malaysia. It was noteworthy as Liu and Wu (2010) described that more than 55 billion people worldwide facing uncontrolled exposure of aflatoxin and the burden of aflatoxin-induced liver cancer still remained unclear. Although it seems impossible to remove and eliminate aflatoxin completely from the food chain, these data are useful to develop plans to control and monitor aflatoxin in the foodstuffs. In fact, interventions could be done to prevent human exposure to aflatoxin and reduce the incidence of aflatoxin-related diseases in Malaysia.

**Table 2:** Risk assessment of aflatoxin exposure in Malaysia.

Dietary AFB_1_ exposure (ng/kg body weight/day)	Estimated liver cancer risk (Cases/100, 000 population/year)	Cancer incidence attributable to dietary aflatoxin (%)	Reference
24.37–34.00	0.61–0.85	12.4–17.3	Chin et al. (2012)
28.81–58.02^a^	0.72–1.45	14.7–29.6	
0.36–8.89	0.03–0.73	0.61–14.9^c^	Leong et al. (2011)
26.2	0.66^b^	13.5^b^	Sabran et al. (2012)
15–140	4.5–42^d^	91–857^c^	Liu and Wu (2010)
10.69	0.27^b^	5.5^b^	Arzandeh et al. (2010)

a Dietary aflatoxin exposure.

b The value was calculated based on formula given by Chin et al. (2012) – (Dietary exposure × 0.025 cancers/100, 000 in Malaysia).

c The value was computed from formula by Chin et al. (2012) – (Estimated liver cancer risk per 10,000 population/incidence rate of liver cancer in Malaysia of 4.9 per 100,000 × 100%).

d Data obtained from estimated annual HCC per 100,000 population for HBsAG-positive.

## FUTURE – MONITORING AND CONTROLLING THE OCCURRENCE OF AFLATOXIN AND INTERVENTION STRATEGIES

It is a very important task to monitor and control the presence of aflatoxin in the human food resources as it involves many aspects. The legislation of law for instance by setting a certain limit can be considered as the “first defense” to prevent aflatoxin exposure to human. This rule was established following the massive outbreak of diseases associated with aflatoxin back in 1960s as mentioned earlier. Prior to that in Malaysia, aflatoxin was not considered to be a significant problem as it was not covered by the Sale of Food and Drug Ordinance 1952. In fact, the ruling on aflatoxin-associated cases was referred from countries such as in UK and USA (Hamid, 1997). However, around 1980s, Malaysia has promulgated stricter standards to prevent the “flow” of aflatoxin in the food chain by the implementation of Malaysia Food Regulation 1985, through the Food Act 1983. Initially, a maximum limit of 35μg/kg for all mycological contaminants was implemented as reported by studies back in 1990s (Hamid, 1997; Abdullah et al., 1998; Ali et al., 1999). Later, it was revised and the new legislation limit was set to 5μg/kg for all mycological contaminants including aflatoxin. Moreover, a limit of 15 and 0.5μg/kg was set for groundnut for further processing and milk products respectively.

With the enforcement of law, it will be meaningless if no proper actions are taken by the public to remove the aflatoxin-contaminated foodstuffs from the line of production. Although Malaysia has joined Codex Alimentarius since 1971 and strengthened the law on the occurrence of aflatoxin in the foodstuffs, it is still believed that some of the contaminated foods might “escape” during the surveillance process and persist in the food chain. A few studies explained that some contaminated foods may be perceived as safe and edible if there are no sign of defects and contaminations (Jolly et al., 2006; Mohd Redzwan et al., 2012b). Since the main route of human exposure to aflatoxin is through the diets, humans can be directly and/or indirectly exposed. Predicated upon that, interventions strategies have been carried out globally. Liu and Wu (2010) classified the strategies into three categories namely agricultural, dietary, and clinical. The agricultural interventions can be applied during pre- or post-harvest. This strategy is considered as the main primary intervention whereas dietary and clinical interventions are regarded as the secondary intervention (Liu and Wu, 2010).

Since then, various research around the globe have come up with measures to limit human exposure to this food contaminant. The use of adsorbents such as activated carbon, hydrated sodium calcium alumino silicate, zeolite, bentonite, and certain clays are beneficial as they are proven to prevent aflatoxin absorption (Denli and Okan, 2006; Thieu and Pettersson, 2008; Gallo et al., 2010). Recently, the use of probiotic has been studied as one the potential adsorbents of aflatoxin in the gastrointetstinal tract. The *in vitro* and animal studies found the potential of certain probiotic lactic acid bacteria in reducing the bioavailability of aflatoxin (El-Nezami et al., 1998; Haskard et al., 2000; Lahtinen et al., 2004; Gratz et al., 2006; Hernandez-Mendoza et al., 2009; Nikbakht Nasrabadi et al., 2013). In fact, probiotics have many beneficial health effects (Oelschlaeger, 2010), thus its use to counteract the toxicity of alfatoxin could be further examined as one the preventive strategies.

## CONCLUSION

Malaysia is a country that is shifting to become a fully developed nation by 2020 and agricultural sector is one of the driving forces for the economy. The occurrence of aflatoxin in the agricultural commodities can be a big loss to the economy and consequently affect the nation. Aflatoxin is dangerous and potent toxin as a number of expert groups have reviewed the health effect of aflatoxin (International Agency for Research on Cancer [IARC], 1993; Joint FAO/WHO Expert Committee on food additives [JECFA], 1998). In Malaysia, reports and publications on the level of aflatoxin in the foodstuffs are abundant. Nevertheless, from the scarce literatures of human exposure to aflatoxin, it is clear that more works need to be done on this very important matter. This is because the information is useful to estimate the magnitude of aflatoxin exposure in Malaysia. On the global scale, a worldwide networking with other nations is essential to gather data and information for harmonization in order to prevent this problem continuing to happen in the future.

## Conflict of Interest Statement

The authors declare that the research was conducted in the absence of any commercial or financial relationships that could be construed as a potential conflict of interest.
